# Canonical Modeling of the Multi-Scale Regulation of the Heat Stress Response in Yeast

**DOI:** 10.3390/metabo2010221

**Published:** 2012-02-27

**Authors:** Luis L. Fonseca, Po-Wei Chen, Eberhard O. Voit

**Affiliations:** 1 Instituto de Tecnologia Quıímica e Biológica, Universidade Nova de Lisboa / Av. da República, Estação Agronómica Nacional, 2780-157 Oeiras, Portugal; Email: llf@itqb.unl.pt; 2 Integrative BioSystems Institute and The Wallace H. Coulter Department of Biomedical Engineering, Georgia Institute of Technology, 313 Ferst Drive, Suite 4103, Atlanta, GA 30332, USA; Email: i98512@gmail.com

**Keywords:** biochemical systems theory, canonical modeling, dynamical model, heat stress, metabolic regulation, multi-scale control, sphingolipid metabolism, systems biology, trehalose

## Abstract

Heat is one of the most fundamental and ancient environmental stresses, and response mechanisms are found in prokaryotes and shared among most eukaryotes. In the budding yeast *Saccharomyces cerevisiae*, the heat stress response involves coordinated changes at all biological levels, from gene expression to protein and metabolite abundances, and to temporary adjustments in physiology. Due to its integrative multi-level-multi-scale nature, heat adaptation constitutes a complex dynamic process, which has forced most experimental and modeling analyses in the past to focus on just one or a few of its aspects. Here we review the basic components of the heat stress response in yeast and outline what has been done, and what needs to be done, to merge the available information into computational structures that permit comprehensive diagnostics, interrogation, and interpretation. We illustrate the process in particular with the coordination of two metabolic responses, namely the dramatic accumulation of the protective disaccharide trehalose and the substantial change in the profile of sphingolipids, which in turn affect gene expression. The proposed methods primarily use differential equations in the canonical modeling framework of Biochemical Systems Theory (BST), which permits the relatively easy construction of coarse, initial models even in systems that are incompletely characterized.

## 1. Introduction

In most cells, a strong temperature increase in the environmental milieu causes a stress response. Much is known about the details of this type of response (e.g., [1]) and yet, we do not have a comprehensive picture of how the response is organized, regulated and coordinated. It is well understood that heat shock proteins are involved, genes up-regulated, signaling mechanisms triggered and metabolic profiles dramatically altered. Some of these changes commence within minutes and some may last for hours after the first exposure to heat. All response processes at the various hierarchical levels of biological organization are crucial, and significant alterations in any of them have the capacity to cause damage and jeopardize survival. The question thus arises of how a cell manages to coordinate this complex, multi-level-multi-scale response. Answering this question is quite challenging, due to the large number and heterogeneity of the involved molecules and the different time scales at which transcription, translation, metabolism, signal transduction, protein turnover, and other physiological processes occur. Because our unaided mind is not equipped to assess the synergisms and antagonisms between many quantitative, dynamic processes with any degree of reliability, the task of answering questions of organization and regulation suggests the use of mathematical models that are at the core of computational systems biology.

Our objective for the work described in this article is the following. We intend to indicate how to translate the known details of a heat stress response into a computational structure that can then be analyzed and interrogated. Upon sufficient diagnostics and validation, this structure, in the form of a systems biological model, is expected to have the capacity of explaining how the response system works under physiological conditions and how it responds, or fails, under extreme adverse conditions. Specifically, the development of such a model must be capable of genuinely addressing the multi-level-multi-scale nature of the stress response system, by accounting for the system dynamics with respect to changes in gene expression and in the temporal profiles in the abundances of mRNAs, proteins and metabolites. A major challenge associated with this task is that any modeling approach must depend on the ability to identify the important components of a system and to omit or simplify what is less relevant and might distract rather than illuminate. A similarly difficult challenge is the appropriate selection of mathematical representations for the governing processes within the system.

In this project, we focus primarily on the metabolic level of the heat stress response in *Saccharomyces cerevisiae*. The advantages of this slice of the biological hierarchy are the following. First, the set of participating metabolites is reasonably contained in size: It consists of only about a dozen metabolites. Second, much, although not all, is known about the regulatory mechanisms affecting the system. Third, at the protein level, only about thirty enzymes, transporters, transcription factors and other proteins are involved and these proteins are encoded by a corresponding number of genes. Thus, the pertinent set of contributors, while being too large for purely intuitive argumentation, is manageable with computational means.

## 2. Cellular Responses to Heat Stress

Throughout evolution, recurring changes in environmental conditions have forced organisms to develop strategies for maintaining a reasonably well-buffered intracellular milieu, which is characterized by a self-regulated steady state and collectively referred to as homeostasis. The strategies for maintaining homeostasis consist of finely coordinated combinations of short-term or long-term adjustments in the cellular state at different levels of the biological hierarchy. The adjustments themselves tend to depend in magnitude on the degree of stress and are typically transient in nature. Thus, cells subjected to more pronounced stresses respond with higher magnitudes and/or longer lasting adjustments. However, once adapted to the stress situation, gene expression and protein levels tend to settle into a new steady state, which is often remarkably similar to the initial, pre-stress steady-state.

Temperature is an interesting stressor as it occurs frequently in nature, is well characterized, and can change rather quickly. It mainly affects two cellular components directly, namely lipids and proteins. DNA is prone to heat-induced denaturation as well, but this effect is of minor relevance for heat stress studies in yeast, because it occurs only at much higher temperatures of about 75–100 °C [2]. Temperature can, however, contribute to increased damage to the DNA molecule, due to reactive oxygen species (ROS) [3]. Lipids are affected by heat with respect to their stiffness and mobility, which in turn modifies the fluidity of membranes and possibly their proper functioning [4]. However, the exact consequences of heat-induced changes on membrane function are not well understood.

Among the various classes of macromolecules, proteins are thus the main facilitators and conduits of a coordinated stress response. Proteins respond to heat with three distinct changes of great importance: Temperature affects their production from mRNAs; their dynamics of degradation or deactivation; and their folding state, which often translates into changes in activity. These direct alterations in proteins lead to changes that secondarily affect other proteins, genes, metabolites, or signal transduction systems. Because of the importance of these changes, we discuss them below in greater detail.

### 2.1. Protein Production

Temperature affects the production of proteins at the level of translation. Yeast cells temporarily cease growing by reducing ribosome and tRNA synthesis, and they also generally reduce transcription [5]. Ribosomal RNAs account for 80% of the total RNA in a growing cell [6]. Therefore, reducing the expression of rDNA should be expected to free some of the resources needed for a faster expression of proteins that are required to overcome heat-induced problems. Conversely, cells under heat stress up-regulate genes that code for proteins and processes of immediate pertinence, including: energy production through carbohydrate and lipid metabolism; metabolite transport; respiration; redox balance and ROS detoxification; cell wall modification; DNA damage repair; as well as protein chaperones that are used for refolding and degradation.

In addition to affecting the translation rate, heat can alter protein synthesis through changes in the stability of mRNAs. Importantly, some mRNAs become more stable during heat stress. Of particular pertinence are mRNAs of genes that are associated with the transcription factors MSN2 and MSN4 [7], which are involved in stress responses (see below). By contrast, the half-lives of mRNAs whose production depends on heat shock factor HSF1 do not change with or during heat stress [7], whereas the mRNA levels of translation-related genes tend to decrease with heat stress [5].

### 2.2. Protein Denaturation and Degradation

Sufficiently high temperatures induce unfolding, denaturation and aggregation of proteins. This change in structure may be reversible or irreversible. Reversible denaturation is corrected by chaperonins, which refold un- or misfolded proteins, whereas irreversibly denatured and aggregated proteins need to be solubilized and are subsequently removed by proteolysis in the proteasome. The processes of complete unfolding, denaturation, and aggregation are typical for higher temperatures.

### 2.3. Partial Protein Unfolding

At lower temperatures, such as 35–40 °C, the first step in the heat response is a passive, partial unfolding of proteins. The resulting changes in protein structure are much milder than for higher temperatures and, in fact, they are used by the cell for regulatory purposes. In particular, proteins of the important sub-group that possesses chemical activity experience changes in their enzymatic activity. This combination of increased catalytic activity, due to the Arrhenius effect, and decreased activity, due to partial unfolding, leads to something like a bell-shaped response curve of enzyme activity as a function of temperature, with the maximum of activity located at the optimal temperature for the specific enzyme. 

The slight unfolding of proteins triggers distinct, secondary cellular responses, which target: (1) transcription factor activation leading to altered physiological processes; (2) gene expression leading to metabolic adjustments; (3) rapid production of protective metabolites; or (4) signaling systems triggering tertiary responses.

**Figure 1:**
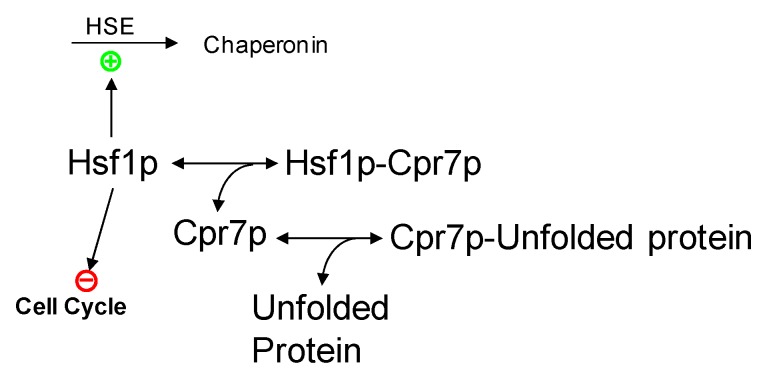
Modeling heat shock factor 1 (HSF1) activation. Free HSF1 protein binds to the heat shock element (HSE) and helps elicit the heat shock response, by inhibiting cell cycle progression and leading to the expression of chaperonins. In the absence of heat-unfolded proteins, the HSF1 protein is kept inactive by the CPR7 protein (Hsf1p-Cpr7p). Under heat stress, the CPR7 protein is sequestered by heat-unfolded proteins, thus releasing its inhibitory effect of HSF1.

*(1) Effects of protein unfolding on transcription factor activation leading to altered physiological processes.* Of particular prominence, the pathway associated with heat shock factor 1 (HSF1) is activated in response to heat (Figure 1). Hsf1p is a transcription factor that recognizes and binds to the heat shock element HSE (5'-NGAAN-3') [8]. Under normal conditions, Hsf1p exists in two states, namely free or bound to HSEs. In both states, Hsf1p is kept repressed through the association with repressor proteins like Cpr7p, Hsc82p, or Sse1p. Heat-unfolded proteins affect the response system through sequestration of these repressor proteins, which thereby permit the activation of Hsf1p [9]. Thus, unfolded proteins free a regulatory protein, HSF1, whose pathway is responsible for the production of protein chaperones, such as HSP82, SSAs and SSBs. HSF1 is also involved in cell cycle regulation and in protein turnover by regulating the expression of the genes UBC4 and CUP1.

**Figure 2:**
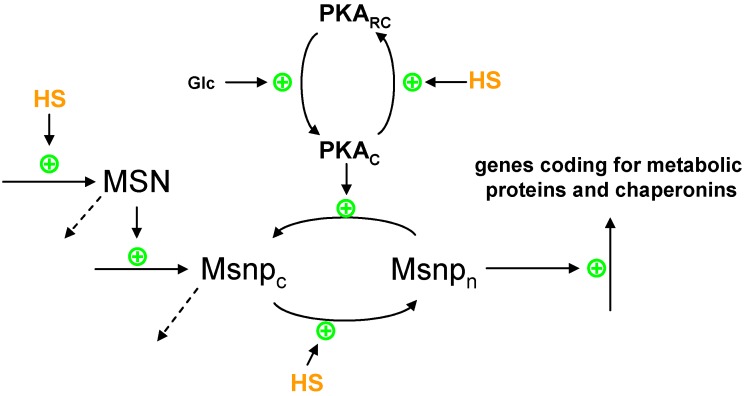
Heat stress affects the localization of MSN protein. The MSN protein (Msnp) is produced from its corresponding mRNA (MSN), which in turn is transcriptionally activated by heat stress (HS). Heat stress promotes a nuclear localization of the MSN protein (Msnp_n_), while active protein kinase A (PKA) (PKA_C_) favors cytosolic localization (Msnp_c_). The activity of PKA is dependent on glucose (Glc) metabolism and heat stress.

*(2) Effects of protein unfolding on gene expression leading to metabolic adjustments.* Heat-induced protein unfolding also targets the zinc-finger transcription factors MSN2/MSN4, which control a large number of genes that appear to be associated with metabolic stress responses (Figure 2). MSN2 and MSN4, collectively called MSN, respond to heat stress and protein kinase A (PKA) in an antagonistic fashion [10]. As long as PKA is active, MSN is kept in the cytosol, where it is inactive. Sufficient heat induction inactivates the PKA pathway and causes MSN to relocate to the nucleus, where it becomes active [11]. Once in the nucleus, the transcription factors bind to specific Stress Response Elements (STRE; 5′-CCCCT-3′) and thereby activate the transcription of downstream genes [12,13]. Genes under the control of MSN code for protein chaperons, proteins involved in protective metabolic pathways (Hxk1p, Tps1p, Nth1p, Gpd1p) and proteins involved in antioxidant defenses (Ctt1p, Sod2p) [9].

Thus, the HSF1 and MSN pathways play different, complementary roles, indicating a division of labor between the two transcription factors. HSF1 controls physiological processes that are temporarily dispensable, such as cell cycle activities, and is essential for the cell’s recovery from short, high-intensity heat shock. By contrast, MSN seems to be primarily in charge of long-term survival at high, but tolerable temperatures [14]. A good review, although not recent, can be found in this reference [9].

One should note that heat affects the regulation of a number of genes that code for enzymes involved in central carbon metabolism. Two modes of action seem to play a role: Some steps are catalyzed by more than one protein paralog, in which case some of the paralogs are heat-inducible while the others are not (Table 1). Additionally, all genes coding for producing and degrading enzymes in some metabolic cycles (e.g*.*, trehalose or glycogen) are up-regulated, which hints at the existence of downstream regulatory processes.

**Table 1:** Differentially regulated protein paralogs (adapted from [1]).

ESR * Genes	Non-ESR* Paralogs	Function
HXK1	HXK2	Hexokinase
GLK1	YDR516C	Glucokinase
PGM2	PGM1	Phosphoglucomutase
PFK26	PFK27	2-phosphofructokinase
FBP26	FBP1	Fructose-2,6-bisphosphatase
GPM2	GPM1, GPM3	Phosphoglycerate mutase
GSY2	GSY1	Glycogen synthase
GLG1	GLG2	Glycogen initiator
GND2	GND1	6-phosphogluconate dehydrogenase
GPD1	GPD2	Glycerol dehydrogenase

* ESR–Environmental Stress Response.

*(3) Effects of protein unfolding on the rapid production of protective metabolites.* Heat-induced protein unfolding, directly affects events at the metabolic level. In particular, temperature alters the activity of several enzymes of the trehalose pathway, thereby leading to the accumulation of the disaccharide trehalose, which protects proteins, membranes and DNA from damage. Intriguingly, heat stress induces a simultaneous increase in the expression of genes coding for both the synthesis and degradation of trehalose, glycogen and fructose-2,6-biphosphate [1]. This increased capacity for production and degradation of intermediates is at first puzzling, and one might be tempted to conclude that it constitutes a futile cycle. However, it rather appears to be evidence of a downstream regulatory mechanism. Such a mechanism can be inferred very nicely from the case of trehalose. Here, the producing enzymes (trehalose 6-phosphate synthase and phosphatase; Tps1p and Tps2p) have activity optima at temperatures of 35–45 °C, whereas the degrading enzyme (trehalase; Nth1p) has its optimum temperature at 30 °C [15]. With this discrepancy in optimal temperatures, very little trehalose is produced at 30 °C, and because trehalase is at its maximum activity, no trehalose accumulates. However, at 40 °C, trehalose production is high and the trehalase activity is reduced by a factor of ~2.4, which causes trehalose to accumulate. Once the temperature returns to normal values, the direct temperature dependence of these enzyme activities allows the cell immediately to degrade all trehalose accumulated at the higher temperature. Not to be wasteful, this degraded trehalose enters glycolysis in the form of two molecules of glucose.

In a slightly different mechanism, glycogen production and degradation are regulated by cAMP dependent phosphorylation: in the presence of glucose, glycogen synthase is activated and glycogen phosphorylase is inactivated; at higher temperatures (35 °C), the glucose effects on these enzymes are amplified [16].

*(4) Effects of protein unfolding on signaling systems triggering tertiary responses.* Heat-induced protein unfolding is expected to influence numerous signal cascades. For instance, an initial cascade of the cAMP-PKA system (see above and Figure 2) is directly affected by heat stress [17].

Another intriguing example is the effect of heat on the activity of enzymes involved in the sphingolipid pathway [18]. We will discuss this system later in more detail but, briefly summarized, it is known that sphingolipids like ceramide and sphingosine-1-phosphate play direct signaling roles in a variety of cell programs [19]. Specifically within the context of stress responses, heat induces changes in the enzyme profile of the biosynthetic pathway, which can lead to a significant alteration in the concentration profile of these lipids. This altered profile, in turn, evokes secondary changes in gene expression. It furthermore causes indirect ripple effects that initially affect the concentrations of other lipids, which again may have their own signaling functions. As a particular example, it was recently shown that heat stress induces an increase in the concentration of phytosphingosine-1-phosphate, which peaks about 10 to 20 min into the stress. The increase in this sphingolipid, in turn, has an effect on numerous other sphingolipid species and also regulates genes associated with cellular respiration, by affecting the HAP transcription factor complex [20].

## 3. Modeling Heat Stress Responses

### 3.1. General Considerations

As indicated in the previous paragraphs, heat induces a number of direct and mediated responses. While these commence more or less immediately when the temperature rises, their dynamics is quite different. As a case in point, the unfolding of proteins is very rapid, and if the protein is an enzyme, the corresponding change in catalytic activity is just as fast. By contrast, alterations in gene expression lead to physiological effects that are delayed by fifteen minutes or more, due to the time it takes to execute transcription and translation.

The human mind tends to have difficulties integrating diverse quantitative information, arising at different time scales, into numerical or even semi-quantitative mental constructs, and this shortcoming suggests the application of computational modeling. Modeling approaches in these situations are challenging as well, again because of differing time scales and because of the heterogeneity of the biological components contributing to the response.

Two generic, successful strategies in such a situation are the separation of time scales and the representation of processes in the format of a canonical model. The separation of time scales consists of focusing on a single time scale while keeping processes at distinctly different time scales constant. For instance, for the short time period where protein unfolding alters the activity of an enzyme, one assumes that changes in gene expression are inconsequential. Their effects will be seen later, but not during the first few minutes of changes in enzyme activities.

The use of canonical representations facilitates the initial model design. These representations, including uni- or multi-variate linear or power-law functions, permit the immediate translation of a dynamic interaction diagram into a symbolic, mathematical construct, which even at this early state allows certain diagnoses and analyses [21,22]. We demonstrate these strategies in the following section, starting with the main transcriptional regulators, MSN2 and MSN4. These are partially redundant, although MSN4, which is inducible by heat stress, is only mildly affected by it [5].

### 3.2. Canonical Modeling

The development of a comprehensive mechanistic model of the transcriptional and translational processes is infeasible with our current modeling technologies, because the detailed physical and chemical events leading to the formation of an intact protein are exceedingly complex. Even within the realm of metabolism, which is much better understood, the choice of a mechanistic model is not without problems. As a case in point, the Michaelis-Menten approximation is often chosen as a default model for enzyme catalyzed reactions, but this rate law is in truth somewhat problematic because its underlying assumptions are not satisfied *in vivo* [23,24]. For instance, the intracellular milieu is certainly not homogeneous and well mixed; the total amount of enzyme is likely to change as a function of time, and a substrate may not exist in much higher concentrations than its enzyme. Thus, one must question whether the Michaelis-Menten representation can be validly used to capture the dynamics of enzymatic processes *in vivo*. Similarly, mass action kinetics is frequently used, but approximating the interactions between several proteins and RNAs in a crowded intracellular environment with an elementary reaction is probably not truly appropriate.

At a very coarse level, the biological complexity and the need for relatively unbiased representations can be tamed to some degree by the use of canonical modeling representations, such as power-law functions, which time and again have been shown to work well for the formalization of complex networks or systems. In particular, these functions are well suited as initial default representations for different types of interactions that are *a priori* ill characterized [25]. The use of power-law functions in such situations is a good compromise that does not impose linearity between components, is mathematically guaranteed to be correct at some nominal operating point, and often provides a reasonable approximation within an acceptable range of concentrations [26]. Due to these features, power-law functions are the central component of Biochemical Systems Theory (BST) [21,24,26,27], which provides a rigorous theoretical framework for modeling and analyzing biological systems.

One great advantage of power-law representations is that the model design step is in principle straightforward: Suppose a process *P* is directly affected only by a substrate *S* and a modulator *M*. Then we know immediately that this process is represented as a function of the type 


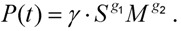
(1)

Here *γ* is a positive rate constant, and the exponents *g*_1_ and *g*_2_ are real-valued kinetic orders, the first of which is positive, because *S* is the substrate, and the second of which is negative if *M* is an inhibitor or positive if it is an activator. The magnitude of each kinetic order reflects the strength of the effect of the variable, with which it is associated, on the process. In fact, if the modulator in Equation (1) has a negligible effect on *P*, its kinetic order *g*_2_ is close to 0, *M* raised to this number is close to 1, and the influence of *M* essentially disappears from the equation.

In the case of heat stress in yeast, power-law functions may be used to represent the overall synthesis of transcripts as well as their degradation. To represent the specific case of a gene under the control of MSN, such as TPS1/2 or NTH1, the nuclear form of the Msn protein is included in the power-law function for gene expression, because it exerts a positive, activating effect (see Figure 2). The dynamics of proteins are formulated in canonical models in a similar manner, namely through overall production and degradation terms. For example, the power-law term for protein synthesis is formulated to depend directly on the abundance of its corresponding transcript.

As a more complex example, but again of the same mathematical format, Equation (2) shows how different factors can be included in a power-law representation (see [28]). In this case, we model a reaction step *F_i_*, in which the enzyme activity depends explicitly on the temperature in the milieu. As before, we include in the representation the substrates (*S_j_*) and modulators (*M_k_*), and account for their respective roles with kinetic orders *h_i,j_* and *hm_i,k_*. We also specify a rate constant *α_i_* and explicitly account for the amount of enzyme, *P_i_*. If we are justified to assume a direct proportionality between enzyme amount and activity, its kinetic order is 1; otherwise a different, more appropriate kinetic order would be included. Finally, *Q_i_* is the direct effect of temperature (*T*) on this enzyme (with reference to 30 °C). It is usually not included in metabolic models, but obviously becomes important for heat-stress studies. Therefore, the power-law formulation of the reaction step reads 


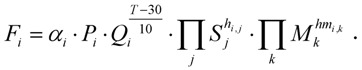
(2)

Further details can be found in [28]. Thus, setting up a dynamic model in a symbolic canonical format is straightforward, because it is clear how different pieces of information are to be converted into components of the mathematical model. The real difficulties arise later, namely in the determination of appropriate parameter values, which are seldom known. However, as a default, experiential values and educated guesses can be employed, at least initially [29]. Upon completion, a canonical model of the transcriptional and translational aspects is expected to simulate the effects of heat stress on the concentrations of mRNAs and their corresponding proteins, at least in a coarse-grained manner.

### 3.3. Parameterization

While the proper translation of a biological phenomenon into a computable structure continues to be an unsolved challenge, it is relatively straightforward to set up initial canonical models in symbolic form, as described before. Yet, achieving the construction of such a symbolic model is only the first step of quantitative model design. A second challenge to be addressed is the identification of appropriate parameter values, and thus the mining of data and kinetic information. Depending on the level of modeling, different types of data and different methods have been proposed, but none of them so far is truly satisfactory [30].

For aspects of heat stress associated with transcription, Gasch *et al.* [5] published a seminal paper that describes numerous transcriptional responses of yeast to environmental changes. The paper is based on data that were made publically available [31] and, among other scenarios, quantifies how most of the transcriptome responds to a temperature jump from 25 °C to 37 °C. Indeed transcript levels are presented for a period of 80 min after the initiation of heat stress. Two further studies [32,33] also induced gene expression patterns under heat stress. Other authors [34,35,36] published complementary datasets for transcript abundances, transcriptional rates and transcript half lives. More recently, Castells-Roca *et al.* [7] published a genome-wide dataset containing mRNA amounts, as well as transcription and decay rates of each mRNA, obtained in a growing culture of yeast cells that were heat stressed by a temperature shift from 25 °C to 37 °C; the data were presented for several time points up to 40 min.

Some data are also available at the proteome level, although these are often not as precise and reliable as for transcripts. For instance, the literature contains accounts of protein amounts, translation rates and protein half-lives, albeit only under control conditions [34,37,38]. Also, more recently, a proteome-wide study characterized the changes triggered by shifting a yeast culture from 24 °C to 37 °C, but this study contains results for only two time points (0 and 30 min) [39]. In principle, these types of datasets render it possible to parameterize the aspects of a multi-level model that are related to transcripts or proteins. 

To refine and extend the parameterization of the metabolic aspects of the model, additional data are needed. Often these are collected from different sources, which sometimes causes problems, due to variations in experimental conditions. Of course, further metabolic studies could be performed under essentially the same conditions that were used to study the transcriptome and proteome, so that a consistent dataset would characterize the three levels of transcripts, proteins, and metabolites. Some kind of standardization has already started to occur in this direction [40], at least for enzyme kinetics.

### 3.4. Modeling Gene Expression and Protein Production

**Figure 3:**
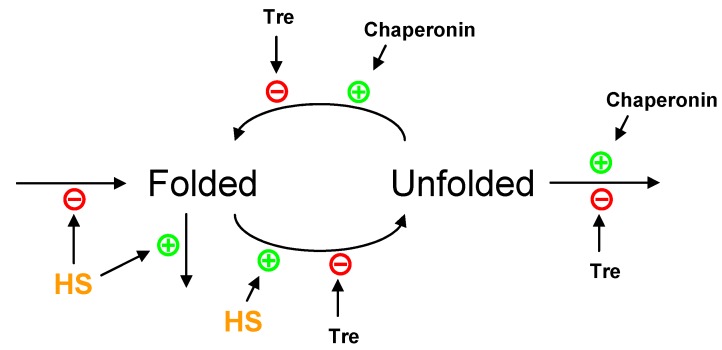
Scheme of the competing forces affecting protein folding and unfolding. Heat stress (HS) causes the unfolding of proteins, while chaperonins promote their refolding. Trehalose functions as a protein stabilizer preventing denaturation and aggregation; however, it also interferes with the proper functioning of heat shock proteins (here represented by Chaperonin) in the refolding of denatured proteins.

We demonstrate the generic modeling approach by beginning at the gene expression level. Of particular importance for heat stress responses are MSN2/4, as discussed before. For simplicity, it is useful to model these two transcription regulators as just one MSN gene or protein. This simplification seems to be supported by their structural and functional similarity. Associated with this transcription factor are a basal level of expression and the provision that heat might slightly increase this expression. As discussed previously, the activity of MSN also depends on protein kinase A (PKA), which itself is affected by cAMP and stress. A recent model [17] integrates these phenomena. It describes the PKA system in great detail and leads to the conclusion that cAMP-PKA and stress may cause an oscillatory shuttling of Msn2p between nucleus and cytoplasm. However, the model does not describe mechanistically or operationally how heat stress changes the localization of the MSN protein. Thus, by adjusting the main concepts of this model to our purposes, one might propose to model the change in localization according to the scheme in Figure 2, where heat stress promotes nuclear localization, whereas activation of PKA favors cytosolic localization. In this approach, PKA is modeled in one of two states, namely, activated (PKA_C_) or inactivated (PKA_RC_). The conversion to the activated state depends on glucose, whereas heat stress inactivates PKA. Once in the nucleus, the Msn protein activates the expression of genes coding for some of the enzymes associated with heat stress (TPS1,2; HXT5; ZWF1; HXK1; GLK1; PGM2; GPM2; GSY2; GLG1; NTH1) and with generic chaperonins that possess refolding functionality (see later and Figure 3). In a canonical model, the qualitative description of the various influences is straightforwardly translated into power-law terms that contain each contributing factor as a variable with an exponent [21,25].

The expression of HSF1 does not seem to change much with heat stress [5], and it is therefore not necessary to model its gene expression. Instead, one considers the total amount of protein as constant and partitions this amount into different activity states. Specifically, HSF1p can exist in three states: free, bound to HSE, or bound to repressor proteins (Figure 1). Hsf1p is kept inactivated by binding to a number of proteins with similar function. Again for the simplicity of an initial, coarse model, one representative for these proteins may be chosen, and a good candidate is Cpr7p. Activation of Hsf1p secondarily induces the expression of chaperonins and inhibits the cell cycle. A mathematical model of these aspects is found in this reference [41].

In order to model Hsf1p activation, we consider a pool of yeast proteins that is prone to heat denaturation and serves the purpose of providing the signal input to the heat stress response (Figure 1). These proteins might be enzymes or structural proteins that tend to unfold at non-optimal temperatures, or they might be intrinsically disordered proteins that are known to have signaling functions [42]. These types of heat-induced effects can be converted into a canonical model where a folded protein controls a heat signaling pathway and where its unfolding triggers—or at least contributes to—a stress response.

As is typical in nature, the ultimate response to a stress situation is the result of a balance between opposing forces. We already discussed the counteracting effects of cAMP-PKA and heat on the localization of MSN2/4 (Figure 2). Another example of the balance of opposing forces is the folding, unfolding, and refolding dynamics of proteins (Figure 3). The disaccharide trehalose protects proteins from unfolding, but interferes with the refolding and degradation of the unfolded protein [43]. By contrast, chaperonins (as representatives of heat shock proteins) promote refolding and facilitate the degradation of unfolded proteins. If these forces are entered into a model, the degradation of unfolded forms has to be balanced with the production of proteins, so that the model may eventually reach a steady state. This production term may be made heat stress sensitive, which is in line with the observation that many transcripts are simply down-regulated under heat stress [5]. At the same time, protein degradation is known to be affected by heat, and inclusion of this effect in the model might improve the functioning of this hypothetical signaling pathway under stress. 

### 3.5. Modeling Specific Metabolic Events under Heat Stress: The Trehalose Cycle

Events at the metabolic level are typically easier to model than at other levels, because specific kinetic information is often available and phenomena like the conservation of mass in reactions provide very valuable constraints that aid the parameter estimation process. As a consequence, several models have been proposed to analyze heat stress and its metabolic effects in yeast and other organisms (e.g., [44,45,46,47,48,49,50,51]). For example, Voit and Radivoyevitch [48] used a canonical modeling approach to study the metabolic consequences of changes in gene expression following heat stress. Although quite simplistic, the model suggested an explanation for the observed heat-induced expression profile, which is quite counterintuitive, with some genes up-regulated many fold, and other genes, even those coding for neighboring reactions, not changed in expression at all. Another study demonstrated the design features of the trehalose pathway with controlled comparisons that identified the role of every regulatory signal at the metabolic level, as well as the observed gene expression patterns [44]. Sorribas and his group refined these types of analyses with sophisticated optimization methods that explained why the observed gene expression patterns are metabolically superior to *a priori* imaginable alternatives [45,46,47,52]. These types of studies have shown that it is indeed possible to infer, with a fairly good degree of confidence, the changes in metabolic states from gene expression or, conversely, the changes in expression profiles from a metabolic model and a set of established physiological criteria based on experimental information.

Earlier studies relied on a possibly significant simplifying assumption, namely that there is a linear correlation between the changes in transcriptomic and proteomic profiles. Maybe more importantly, these approaches ignored the direct temperature effects on enzyme catalysis. A more recent model [28] takes these aspects into account. In particular, this work joins two dynamic sub-models that represent different time scales and shows that canonical models, using power-law functions (as in Equations (1) and (2)), can be constructed from experimental data in a top-down manner. The first sub-model simulates the time-dependent protein profiles from the network of interactions between transcripts and proteins, while the second sub-model is a metabolic model that is capable of simulating time-dependent metabolic profiles based on the amounts of enzymes catalyzing each step, which are supplied from the first sub-model.

The main focus of this joint model is the enormous accumulation of trehalose in response to elevated temperature. Interestingly, targeted experimental analyses demonstrated that naïve and heat-adapted cells respond in a qualitatively similar, but quantitatively very different manner. In particular, when cells are exposed to heat during their early exponential growth phase, later heat stress leads to almost ten times the amount of accumulated trehalose in comparison to naïve cells [28]. To analyze this phenomenon, we set up a model in the following fashion. We allowed the naïve and heat adapted cells to express different amounts of the enzymes that catalyze each metabolic step in the trehalose pathway. This strategy accounted for the fact that cells exposed to heat during growth had the opportunity to increase gene expression and thereby the abundance of pertinent mRNAs and proteins. Our experimental time series data even allowed us to quantify these changes numerically. Additionally, because these data were obtained under two different temperatures (30 °C and 39 °C), we were able to quantify some of the direct effects of temperature on the enzymatic activities, while the temperature dependence of the activities of the trehalose related enzymes was obtained from literature [15]. By combining this type of a metabolic model with a model capable of predicting changes in enzymes, brought about by the heat stress response (see Equation (2)), it is possible to obtain a model that predicts metabolic responses and the cell’s adaptation to heat stress exposure quite well. Also, by combining the two sub-models, we are in a position to close the trehalose loop, since trehalose production is accounted for in the metabolic sub-model, while its effects as protein stabilizer are modeled in the transcriptomic/proteomic sub-model. Thus, this modeling strategy allows us to grasp how the various components interact synergistically to elicit, regulate, and sustain an appropriate heat stress response.

### 3.6. Modeling Specific Signaling Events under Heat Stress: The Role of Sphingolipids

**Figure 4:**
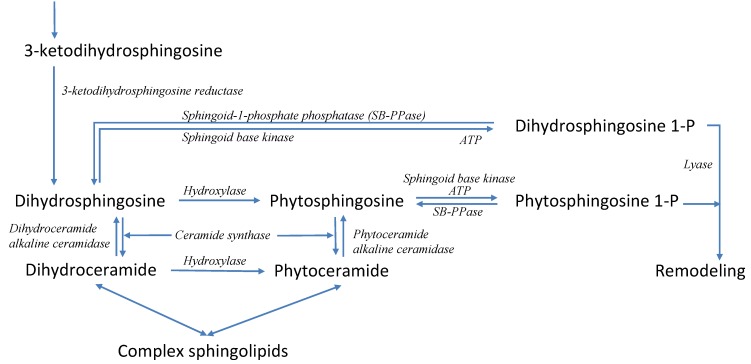
Simplified pathway system of sphingolipid biosynthesis and usage in yeast. Sphingolipid biosynthesis is initiated with the condensation of palmitoyl-CoA and serine, leading to 3-ketodihydrosphingosine, which is quickly converted into dihydrosphingosine, the first simple sphingolipid in the pathway. Dihydrosphingosine is the starting point for five other key sphingolipids, namely dihydroceramide, dihydrosphingosine 1-phosphate, phytosphingosine, phytoceramide, and phytosphingosine 1-phosphate, which regulate each other’s production and degradation extensively. The system has two exit routes. One is the formation of complex sphingolipids, which become parts of membranes, and the other is the remodeling pathway, which recycles sphingolipids. When heat stress is applied, the six key sphingolipids exhibit strong dynamic changes in concentration. Recordings of these responses over 30 minutes following a shift in temperature serve as our time course data. Pertinent enzymes are shown in italics. Further details are available in [55,56,57,58].

Sphingolipids form a specific class of lipids that are crucial components of membrane microdomains, called lipid rafts, and furthermore play distinct, important roles in the regulation of cellular stress responses, differentiation, proliferation, apoptosis and other fundamental cell functions [19]. Interestingly, evidence has implicated sphingolipids in the coordination of the heat induced expression of genes under the control of the MSN proteins [53]. Sphingolipids have also been shown to be necessary for efficient translation initiation during heat stress [54]. Although the pathways of sphingolipid biosynthesis and degradation have been characterized over the past decades in some detail (see Figure 4), the collective functioning of the pathway is still puzzling, mainly because the unaided human mind is unable to integrate its many components and their regulation in a quantitative manner. Therefore, with the aid of modeling and computational techniques, we set out to characterize the pattern(s) that control the yeast sphingolipid pathway under heat stress.

We began by developing, over the span of a decade, a series of computational models [55,56,57,58]. Formulated within BST, the core model contains about 65 variables. The model was tested and validated, and it appears that it captures many dynamic features of the sphingolopid pathway quite well.

**Figure 5:**
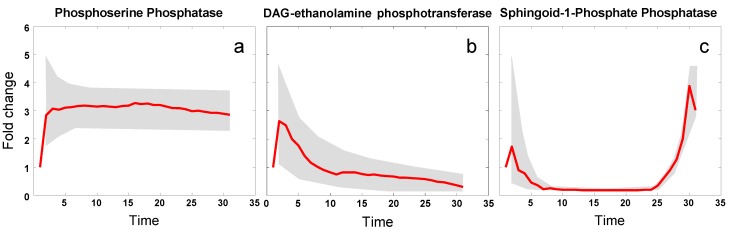
Examples of three classes of heat-induced changes in enzyme activities within sphingolipid metabolism. Heat stress causes the activities of: phosphoserine phosphatase to increase (**a**); diacylglycerol (DAG) ethanolamine phosphotransferase to decrease (**b**); and sphingoid-1-phosphate phosphatase to decrease initially and subsequently to increase substantially, presumably due to gene expression that begins to increase as soon as the temperature rises (**c**). Grey bands indicate ranges of responses, while red lines show average trends.

An ongoing goal of relevance here is the identification of enzymes that are affected in the pathway during a response to heat stress. The analysis, whose technical details will be presented elsewhere, is based on time course data of six key sphingolipids, measured at 0, 5, 10, 15, 20, and 30 min after the beginning of heat stress (39 °C). We smoothed these data with splines and then applied a piecewise optimization approach to estimate the dynamically changing profiles of all enzymes within the sphingolipid pathway, by minimizing the distance between the smoothed sphingolipid data and the simulation results at each time point from 0 to 30. Using a randomization scheme for the initial algorithmic settings, we generated 100 sets of dynamic adjustments in enzyme activities that led to metabolite concentration trends consistent with observations. The overall result thus consisted of a band for each enzyme activity, within which about 90% of all solutions laid, as well as the average trend in each enzyme activity (Figure 5). Details of this analysis will be shown elsewhere.

The results are quite intriguing in detail, because they reveal the balance of three forces acting, on the enzymes, induced by heat: Increased activity according to an enzyme’s Q_10_ value, as alluded to in Equation (2); diminished activity due to partial protein unfolding, an altered half-life of the corresponding protein and/or mRNA, and/or a reduced production; and change in enzyme activity due to gene expression. As an example for the first category, the activity of phosphoserine phosphatase increases about three-fold and remains at this activity level for at least 30 min (Figure 5a). An example of the second category is diacylglycerol (DAG) ethanolamine phosphotransferase, whose activity was inferred to decrease, after a brief initial increase according to its Q_10_ (Figure 5b). Sphingoid-1-phosphate phosphatase falls into the third category (Figure 5c). Initially its activity drops quickly, but after about 25 min not only recovers but increases well over its baseline activity. Of note is that these results were extracted from the concentration time series data and the dynamic model strictly by computational means and without additional information.

## 4. Conclusions

In the past, the effects of heat stress adaptation in the central carbon metabolism of yeast cells have been modeled by forward approaches, that is, by constructing models from their components and subsequently assessing the effects of heat. Several of these studies were ultimately based on a steady-state metabolic model of glycolysis published by Curto *et al.* [59]. After extensions and adjustments, these models were subjected to what-if simulations and to validation tests of the consistency between model predictions and known information about the physiology of heat stress adaptation. An example of this strategy is [48]. The Sorribas group [45,46,47,52] improved on these early studies by developing rigorous optimization methods to explore the space of reasonable combinations of gene expression profiles and study the feasibility of each profile according to *a priori* established criteria.

Accounting for the fact that enzyme activities are likely to change simply due to the increased temperature, we performed targeted experiments to generate new metabolic data [28]. They consisted of time series measurements of naïve and heat-stressed cells and allowed us to construct and parameterize a new dynamical model capable of inferring, in an inverse modeling approach, the likely enzyme expression profiles from our data. With this approach we were able to show that the inferred enzyme expression profile is similar to what is known to happen to the gene expression profile *in situ*. In our sphingolipid work, which was described briefly here and will be presented elsewhere in detail, a computational optimization analysis was able to infer changes in enzyme profiles following a shift in heat. Intriguingly, the model analysis, without manual intervention or human curation, identified enzymes that likely respond to heat simply according to their direct sensitivity to temperature and others that seem to respond to changes by targeted gene expression. Together, these two studies indicate how heat induces changes in proteins, which are transduced in parallel, directly or via lipid signaling, to the level of gene expression, which in turn facilitates a well-coordinated heat response and to longer-term metabolic adaptations.

**Figure 6:**
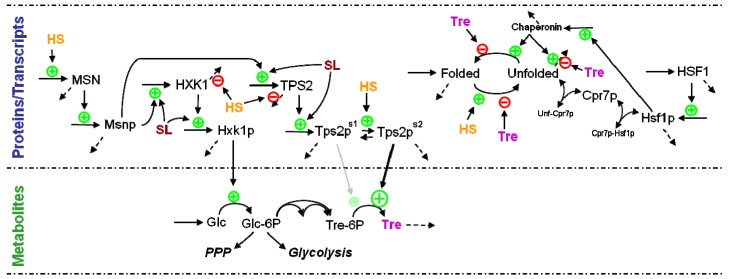
Schematic overview of the multi-scale regulatory model of the heat stress response. Heat stress (HS) increases the expression of the transcription factor MSN, which in turn regulates genes that code for enzymes of central metabolism that are involved in the cell’s protection against protein unfolding (e.g., hexokinase and trehalose-6P phosphatase). Additionally, the expression of these MSN-induced genes is coordinated by sphingolipids (SL), whose pathway dynamics is directly affected by heat. Furthermore, heat stress increases the half-lives of transcripts, as well as the activity of Tre-6P phosphatase. The presence of heat-unfolded proteins releases the inhibitory effects of Cpr7p on Hsf1p, thus up-regulating the expression of HSF and of chaperonins, which in turn promotes the refolding of unfolded proteins. Trehalose acts as a protein stabilizer. Dashed arrows depict degradation processes.

While many studies on heat stress responses are available in the literature, it seems that we are approaching a situation where many experimental observations can be merged successfully into a computational construct that combines the direct and indirect effects of heat, for instance on partial protein unfolding, and on gene expression, metabolic state, and cellular physiology. The next steps toward such a computational construct will include more complete models of the gene regulatory network at the heart of the long-term heat response. Such a model (Figure 6) will have to integrate much diverse, and often qualitative, information on the connectivity and regulation of gene expression, and combine this information with time series data, characterizing gene and protein expression profiles, rates of transcription, and half-lives of transcripts obtained in yeast cells growing under heat adaptation. At present, some of the required datasets for such a comprehensive model are available for control and stressed cells, but sufficient time series data of protein production (rate of translation) and protein half-lives in cells under heat stress are still lacking. The reward of combining, in a fully dynamical model, aspects of gene regulation, protein changes, metabolic state changes, and signaling events will be a much improved understanding of a paradigmatic control task in biology.

Heat stress responses constitute a particularly well studied multi-scale system, and few other stress systems have received similar attention. Nonetheless, other stresses, in particular within the context of disease, have been studied with methods of canonical modeling (e.g., [60,61,62,63,64]). In many cases of such modeling efforts, the focus was on a single level (such as metabolism), and we are only now slowly addressing truly multi-level-multi-scale systems, because data at several levels and scales are becoming available and the modeling community has progressed considerably in recent years. Nevertheless, because it seems presently infeasible to capture the essence and details of complex stress or disease systems in one grand modeling effort, it appears to be useful to begin with coarse, mesoscopic models of intermediate complexity and to use these, on the one hand, for exploring features of natural system design and, on the other hand, to move toward realistic disease simulators [65].
